# Photocurable Epoxy Acrylate Coatings Preparation by Dual Cationic and Radical Photocrosslinking

**DOI:** 10.3390/ma14154150

**Published:** 2021-07-26

**Authors:** Paulina Bednarczyk, Karolina Mozelewska, Małgorzata Nowak, Zbigniew Czech

**Affiliations:** Department of Chemical Organic Technology and Polymeric Materials, Faculty of Chemical Technology and Engineering, West Pomeranian University of Technology in Szczecin, Piastów Ave. 42, 71-065 Szczecin, Poland; karolina_mozelewska@zut.edu.pl (K.M.); gosia.nowak.zut@gmail.com (M.N.); psa_czech@wp.pl (Z.C.)

**Keywords:** epoxy acrylates, coatings, photopolymerization, photoinitiators

## Abstract

In this work, epoxy acrylate resin (EA) based on the industrial-grade bisphenol A-based epoxy resin (Ep6) and acrylic acid (AA) has been synthesized in order to develop hybrid resin comprising both epoxide group and reactive, terminal unsaturation. Obtained epoxy acrylate prepolymer was employed to formulate photocurable coating compositions containing, besides the EA binder, also cationic or radical photoinitiators. Hence, when cationic photoinitiators were applied, polyether-type polymer chains with pending acrylate groups were formed. In the case of free radical polymerization, epoxy acrylates certainly formed a polyacrylate backbone with pending epoxy groups. Owing to the presence of both epoxy and double carbon–carbon pendant groups, the reaction product exhibits photocrosslinking via two distinct mechanisms: (i) cationic ring-opening polymerization and (ii) free radical polymerization. Therefore, photopolymerization behavior of synthetized hybrid resin with various photoinitiators was determined via photo-differential scanning calorimetry (photo-DSC) and real-time infrared spectroscopy (RT-IR) methods, and properties of cured coatings were investigated. The performance of the following type of photoinitiators was tested in the cationic photopolymerization: diaryliodonium cations or triarylsulfonium cations, and the following type of photoinitiators were used to induce free radical photopolymerization: α-hydroxyketones, acylphosphine oxides, and their mixtures. Lastly, the basic physicomechanical properties of cured coatings, such as tack-free time, hardness, adhesion, gloss, and yellowness index, were evaluated. Some structural factors and parameters of cationic and radical photoinitiators and photopolymerization mechanisms affecting the epoxy acrylate hybrid coatings performance are discussed.

## 1. Introduction

The polymerization of multifunctional monomers or oligomers by UV curing has become a widely used technology that has found many industrial applications due to its unique advantages. The coating industry is the most important application of UV curing technology and used to protect the surface of materials with quick-drying varnishes or printing inks. Other uses include manufacturing solder mask, stereolithography, and microelectronic photoresists. In addition, quick-setting adhesives, coatings, sealants, and the production of composite materials are just some of the many applications of photopolymerization [1,2,3]. So many applications result from the high speed of curing by UV radiation and the high efficiency of the process. This solution also exhibits a few other advantages; it is environmentally friendly with the possibility of adjusting parameters (from brittle to very flexible films), curing at ambient temperature, and adjusting the properties of the final product to the needs [4,5]. Compared to traditional coatings (e.g., solvent-based coatings), UV-curable coatings provide energy savings and low volatile organic compounds [6,7,8]. This is believed to be the most effective method to rapidly convert a solvent-free liquid resin into a solid polymer at ambient temperature. Under intense lighting, the cross-linking polymerization of the resins is intense, sometimes within a fraction of a second, creating a dense three-dimensional polymer network that exhibits excellent resistance to organic solvents, chemicals, and heat [4,6].

In the photopolymerization process, the type of monomer and photoinitiator are of great importance, because the compositions can be polymerized using a free radical or cationic photopolymerization mechanism [7,8]. Currently, free radical photopolymerization is used most often due to the large assortment and low cost of free radical monomers, photoinitiators, and additives to set the final properties, with the possibility of combining them into products with different properties. In turn, the disadvantage of these systems is the inhibition of free radical polymerization by oxygen [9,10]. Cationic photopolymerization is an alternative polymerization method in the production of cured coatings. The cationic process is not inhibited by oxygen. Compared to free-radical photopolymerization, it does not stop immediately after the radiation is turned off, but occurs from darkness, sometimes slower, which is referred to as dark curing [11,12,13]. These types of features are important when uneven and complex-shaped coatings are cured. In addition, cationic photopolymerization only leads to low shrinkage (e.g., for epoxy coatings around 5%). In turn, many hydrogen bonds are formed during the process, which leads to an increase in adhesion between the material and the coating [14]. Curing coatings with cationic photopolymerization is often used for monomers that cannot be photopolymerized in a free radical manner, e.g., vinyl ethers and epoxides [15,16].

To overcome the limitations of acrylates and epoxides, a product can be synthesized based on acrylate and epoxy monomers. In this case, the monomers can polymerize by various mechanisms, and the resulting products combine the properties of both acrylates and epoxides, making them suitable for various applications [17]. Epoxy acrylates are characterized by flexibility, hardness, thermal resistance, and non-yellowing. In their case, the epoxy backbone increases strength and flexibility during curing, while carbon–carbon and ether bonds improve chemical resistance. Moreover, due to their low cost, excellent chemical and corrosion resistance, and favorable mechanical properties and good processability, they are used in many areas of the industry [18,19]. Poor exterior durability and high viscosity are the main disadvantages associated with these materials. The use of UV and thermally curable epoxy acrylate prepolymers has increased in recent years. They are used as varnishes, wood coatings, or lithographic paints. They are popular in other areas as well: vacuum metalizing base coatings, video disc coatings, and adhesive laminates [20,21,22].

In this contribution, the modification of epoxy resin with methacrylic acid, allowing the obtainment of a hybrid polymer containing both epoxy and acrylic groups, has been reported. Subsequently, photoreactive coating compositions were prepared from the obtained epoxy acrylate and cured using UV radiation. The samples were tested for the kinetics of the process and the properties of the cured coatings. Both the cationic and free radical photopolymerization of synthetized EA have been investigated by the photo-DSC and RT-IR method, and the suitability of various photoinitiators for photopolymerization of the hybrid systems using a UV lamp is evaluated.

## 2. Materials and Methods

### 2.1. Materials

The industrial-grade bisphenol A-based epoxy resins were purchased from “Organika-Sarzyna” S.A., Nowa Sarzyna, Poland. The following epoxy resins were used: Epidian 6^®^ (Ep6), with epoxide number of 0.51–0.54 mol/100 g and viscosity ranging from 10,000 to 15,000 mPa·s at 25 °C, and acrylic acid (AA), stabilized, with the purity of 99.5%, supplied by Acros Organics, Geel, Belgium. Triphenylphosphine (PPh_3_), Apollo Scientific, Bredbury, UK, was used as a catalyst in the reaction between Ep6 and AA, while hydroquinone (HQ, Acros Organics, Geel, Belgium) was used as a polymerization inhibitor. All chemicals were employed as received.

The following titration reagents and indicators were used: glacial acetic acid, toluene, potassium hydroxide standard solution 0.1 M in ethanol (KOH), and crystal violet purchased from Chempur (Piekary Slaskie, Poland), chloroform form P.P.H. Stanlab (Lublin, Poland), ethyl alcohol from Avantor (Gliwice, Poland), tetraethylammonium bromide provided by Acros Organics (Geel, Belgium), perchloric acid standard solution 0.1 M in glacial acetic acid supplied by Fischer Chemicals (Zurich, Switzerland), and Phenolophtalein 1% in ethyl alcohol solution from Eurochem BGD (Tarnów, Poland). All chemicals were analytical grade and were used as received.

**Figure 1:**
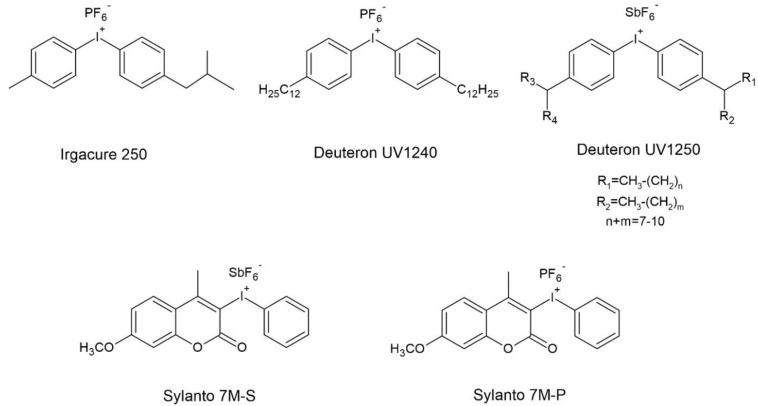
Structure of the cationic photoinitiators based on diaryliodonium cation.

**Figure 2:**
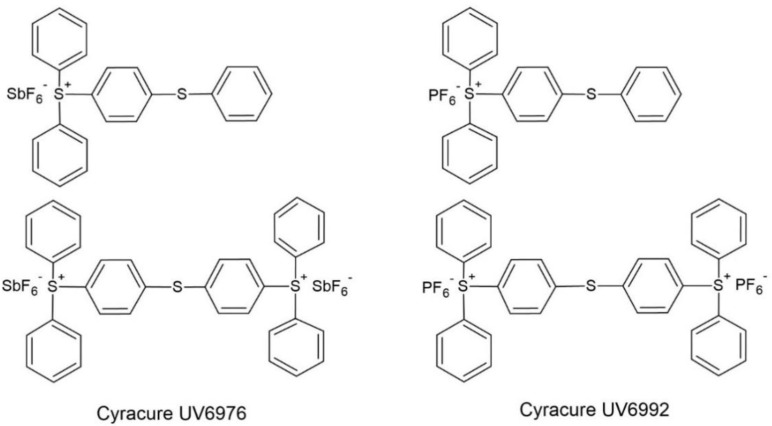
Structure of the cationic photoinitiators based on a triarylsulfonium cation.

The diaryliodonium and triarylsulfonium cation-based photoinitiators were selected from the many cationic photoinitiators available. The following photoinitiators based on the diaryliodonium cation were used to induce cationic photopolymerization of hybrid resin: (4-methylphenyl)[4-(2-methylpropyl)phenyl]iodonium hexafluorophosphate (Irgacure 250) from BASF (Ludwigshafen, Germany), bis(dodecylphenyl)iodoniumhexaflouroantimonate in propylene carbonate (Deuteron 1240) and bis-((C10-C13)alkylphenyl)-iodoniumhexafluoroantimonate (Deuteron 1250) from Deuteron (Achim, Germany), (7-methoxy-4-methylcoumarin-3-yl)phenyliodonium hexafluoroantimonate (Sylanto 7M-S) and (7-methoxy-4-methylcoumarin-3-yl)phenyliodonium hexafluorophosphate (Sylanto 7M-P) from Synthos S.A. (Oświęcim, Poland). The following photoinitiators were selected from the cationic photoinitiators based on the triarylsulfonium cation: mixture triarylsulfonium hexafluoroantimonate salts (Cyracure UV6976) and mixture triarylsulfonium hexafluorophosphate salts (Cyracure UV6992) from Dow (Frankfurt, Germany). Structures of the cationic photoinitiators are shown in Figure 1 and Figure 2.

**Figure 3:**
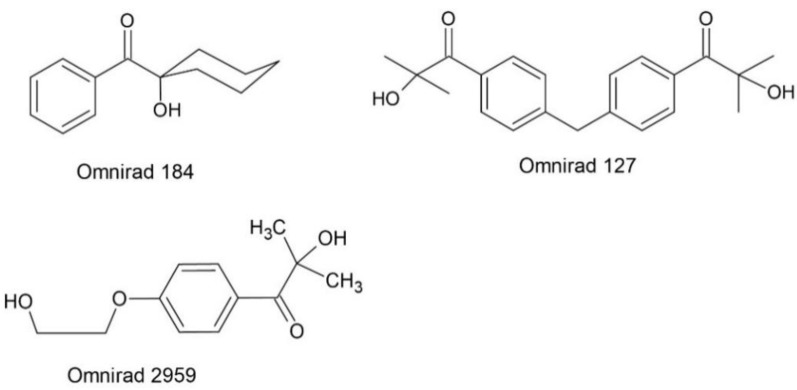
Structure of the free-radical photoinitiators α-hydroxyketones.

**Figure 4:**
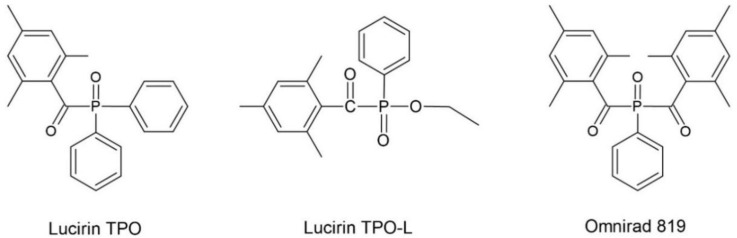
Structure of the free-radical photoinitiators–acylphosphine oxides.

**Figure 5:**
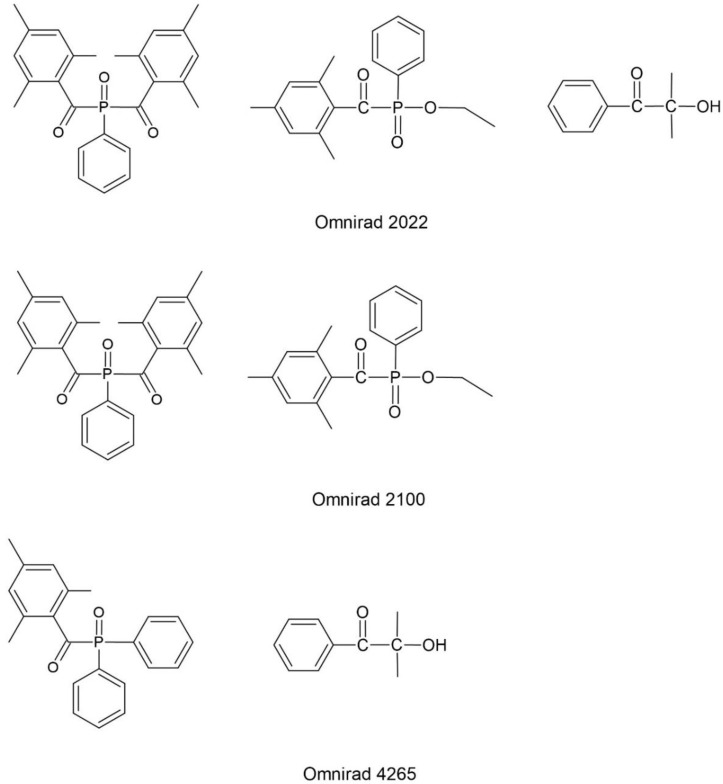
Structure of the free-radical photoinitiators–mixtures.

As free radical photoinitiators for hybrid monomers, photoinitiators were used, which were divided into α-hydroxyketones, acylphosphine oxides, and their mixtures. The following compounds were selected from the photoinitiators with the structure of α-hydroxyketones: 1-hydroxycyclohexyl phenyl ketone (Omnirad 184), 2-hydroxy-1-{4-[4-(2-hydroxy-2-methyl-propionyl)-benzyl]-phenyl}-2-methylpropan-1-one (Omnirad 127) and 1-[4-(2-hydroxyethoxyl)-phenyl]-2-hydroxy-methylpropanone (Omnirad 2959) from IGM Resigns (The Netherlands). The structures of the free-radical photoinitiators α-hydroxyketones are collected in Figure 3. The following were selected as acylphosphine oxides: 2,4,6-trimethylbenzoyl-diphenyl phosphine oxide (Lucirin TPO), (ethyl-2,4,6-trimethylbenzoylphenylphosphinate) (Lucirin TPO-L) from BASF (Germany) and bis(2,4,6-trimethylbenzoyl)-phenylphosphineoxide (Omnirad 819) from IGM Resigns (Netherlands). The structures of the free-radical photoinitiators–acylphosphine oxides are collected in Figure 4. The following were selected as mixtures of photoinitiators: a blend of 2-hydroxy-2-methylpropiophenone phenyl bis(2,4,6-trimethylbenzoyl)-phosphine oxide and ethyl phenyl(2,4,6-trimethylbenzoyl) phosphinate (Omnirad 2022), a blend of ethyl phenyl(2,4,6-trimethylbenzoyl) phosphinate and phenyl bis(2,4,6-trimethylbenzoyl)-phosphine oxide (Omnirad 2100) and a blend of 2, 4, 6-trimethylbenzoyl-diphenyl-phosphine oxide and 2-hydroxy-2-methyl-1-phenylpropanone (Omnirad 4265). All photoinitiators mixtures used were from IGM Resigns (Netherlands). The structures of free radical photoinitiators–mixtures are presented in Figure 5.

It has long been known that the effectiveness of a photoinitiator depends on matching the absorption spectrum with the emission spectrum of the light source. It is important that the maximum of the absorption peak is in the range of the lapa emission. Table 1 shows the maximum absorption peaks of the tested photoinitiators.

### 2.2. Synthesis of Epoxy-Scrylate Pre-Polymer

**Figure 6:**
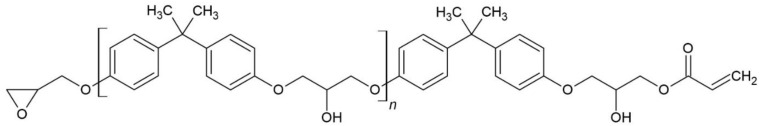
Structure of epoxy acrylate prepolymer.

Epoxy acrylates (EAs) were obtained by the addition of acrylic acid to epoxy resin. The synthesis was carried out in a 250 mL three-neck glass reactor (equipped with a thermometer, a condenser, a nitrogen inlet, and a mechanical stirrer), into which bisphenol A type epoxy resin (Epidian 6) was introduced. Then, hydroquinone (0.0075 wt.% based on total batch weight) was transferred into the reactor as a radical scavenger at room temperature. Lastly, acrylic acid (0.5 mol relative to resin epoxy value) and catalyst–triphenylphosphine in the amount of 0.8 wt.% (relative to the mass of AA) were added. The reaction mixture was heated to 70 °C with vigorous stirring (120 RPM) using an oil bath. Once the homogeneous mixture was obtained, the temperature was raised to 90 °C, and the reaction was carried out for 4 h in a nitrogen atmosphere, with stirring. At room temperature, the as-prepared EA appears as colorless, transparent, viscous liquids. The reaction product is presented in Figure 6. The epoxy acrylate pre-polymer obtained and analyzed in the present work will be denoted as EA-FI, depending on the type of applied photoinitiator.

### 2.3. Characterization Methods

The infrared spectra acquired with a Thermo Nicolet 380 FT-IR spectrometer. Sixteen scans were averaged for each sample in the range of 4000–400 cm^−1^ at room temperature. The non-volatile-matter content (NV) was evaluated thermogravimetrically, using moisture analyzer MAX 60/NP (Radwag, Poland), according to the ISO 3251:2019 standard. Partial acid values (PAVs) were determined by colorimetric titration according to EN ISO 2114:2000 standard. Epoxy equivalent (EE) was determined by means of colorimetric titration, according to the EN ISO 3001:1999 standard. The viscosity tests were carried out using a cone-plate viscometer LAMY RM-100 plus CP 2000. The test methods were described in detail in our previous article on epoxy acrylate prepolymers [23]. The molecular weight studies were performed in tetrahydrofurane with a liquid chromatograph LaChrom system, RI Detector L-7490 and LaChrom UV Detector L-7400 from Merck-Hitachi, equipped with a PLgel 106 Å column from Hewlett-Packard.

### 2.4. Preparation of Coating Compositions and Cured Films

The coating compositions have been formulated using epoxy resin or synthesized epoxy acrylate and 3 wt.% of photoinitiator (cationic or radical). The components were stirred together under dark conditions until a homogeneous mixture was obtained. Subsequently, the curing solution was applied to the glass substrates by means of a gap applicator (120 µm). The polymeric film was cured under a light source (UV lamp, Aktiprint-mini 18-2, type: UN50029, Technigraf GmbH) at room temperature and irradiated under UV light with an intensity of 200 mW/cm^2^ to dryness.

### 2.5. Characteristics of the Photopolymerization Process and Properties of Cured Coatings

The UV-curing process of epoxy or epoxy acrylate varnishes was isothermally monitored (25 °C) for 15 min by means of photo-DSC apparatus (Q100, TA Instruments, New Castle, DE, USA) equipped with UV light emitter Omnicure S2000 (Excelitas Technologies, Waltham, MA, USA). The spectrum range of the lamp is 320–500 nm. The OmniCure S2000 is equipped with a 200-W UV lamp. This allows the generation of UV light with a power of 200 mW/cm^2^. A polymerization solution was composed of epoxy resin (Ep) or epoxy acrylate (EA) prepolymer and 3 wt.% of photoinitiator.

Fourier transform infrared spectra (FT-IR) were obtained on a Nicolet iS5 instrument. The resolution is 4 cm^−1^ and the scanning range is 400–4000 cm^−1^. The recording interval of the spectrum was 10 s. Series real-time IR (RT-IR) was used to determine the conversion of epoxide groups or acrylic double bonds. More importantly, this spectroscopic technique permits in situ monitoring of the chemical processes via mimicking the disappearance of the characteristic bands of the reactive monomer subjected to UV exposure [24]. The mixture of Ep or EA and an initiator was placed in a mold made from glass slides and spacers of 15 mm in diameter and 1.2 in thickness. The samples were placed in the compartment of a Fourier transform infrared spectrometer and were simultaneously exposed to a UV radiation source (mercury UV lamp, 36 W, 280–400 nm, 10 mW/cm^2^) and an IR analyzing light beam. The absorbance change of the epoxide group (C–O) and acrylate double bond (C=C) peak area was correlated to the extent of polymerization. The degree of conversion (DC) can be expressed by the following relations: DC (%) = (A0 − At)·100/A0, where A0 is the initial peak area before irradiation and At is the peak area at time t. The photopolymerization rate (Rp) was calculated by the following relations: Rp = dDC/dt, where t is the time of irradiation [25].

The following tests were performed in order to evaluate the mechanical properties of cured coatings: tack-free time, pendulum hardness test, adhesion, gloss, and yellowness index. Tack-free time was measured as a surface cure time according to ISO 9117. It is the time at which the coating is deemed to be properly adhered to and achieves the final technical parameters. The hardness of coatings was tested using Persoz pendulum hardness on the glass substrate (TQC Sheen, Capelle an den Ijssel, The Netherlands) according to ISO 1522 standard. According to the PN-EN ISO 2409 standard, the adhesion to the glass substrate was determined (cross-cut method; BYK, Wesel, Germany). Gloss was measured by spectrometer GLS (SADT Development Technology Co. Ltd., Beijing, China) according to ASTM D523. The yellowness index is a number calculated from spectrophotometric data that describes the change in color of test samples. This parameter was measured according to ASTM E313 using precision colorimeter NH-145 (3NH Technology Co. Ltd., Shenzhen, China).

## 3. Results

### 3.1. The Approach to the Development of the EA Prepolymer Synthesis Parameters

**Figure 7:**
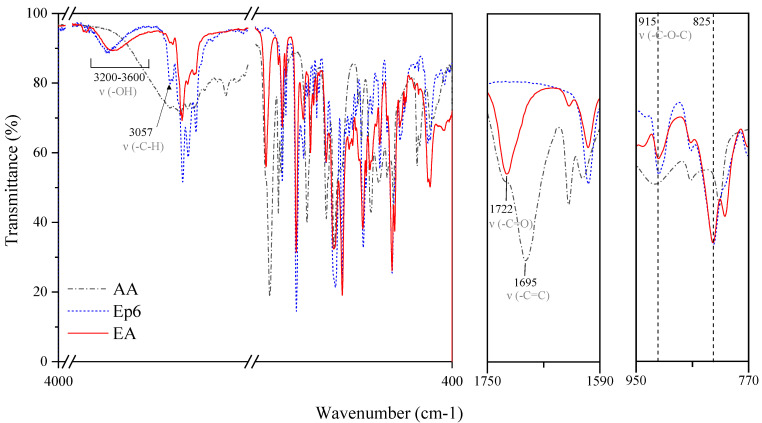
FT-IR spectra of epoxy resin, methacrylic acid, and EA prepolymer.

The addition reaction of AA to the epoxy resin was carried out in order to obtain a hybrid resin containing both epoxy and acrylic groups in the molecule, which will be used in the further part of the research to monitor the course of cationic and radical photopolymerization with the use of various photoinitiators. Herein, the process parameters were developed based on a literature survey [26,27,28,29,30] and several preliminary runs carried out at different reaction temperatures and catalyst doses. The progress of the reaction was monitored by FT-IR spectroscopy. In particular, the absorption bands corresponding to the vibrations of carboxyl and epoxy groups are of special interest to evaluate the extent of reactions in the investigated system. In fact, from Figure 7, it can be observed that in the EA prepolymer, the intensity of the IR absorption bands arising from –C–H (3057 cm^−1^), C–O (914 cm^−1^), and C–O–C (825 cm^−1^) stretching vibrations of oxirane groups [31,32,33] decrease, thus confirming that the ring-opening of epoxy groups took place. Furthermore, one can note that the peak at 1695 cm^−1^ originating from the carbonyl stretching mode in AA [34,35] becomes less intense as a result of the reaction and finally indistinguishable. Meanwhile, an additional absorption peak corresponding to C=O stretching vibrations develops at 1722 cm^−1^, thereby suggesting that new bonds occur due to the reaction of carboxyl groups of AA and epoxide groups of Ep6, i.e., ester bonds. This agrees with the previous studies reporting the shift of the C=O band after the esterification reaction [36]. Lastly, the progress of ring-opening reactions between epoxide groups and AA is highlighted by an increase in the intensity of broadband occurring in the range of 3600–3200 cm^−1^, due to –O–H groups’ evolution [17,33,37] resulting from the epoxide ring-opening.

The product of reaction was also characterized for the non-volatile-matter content (NV), partial acid value (PAVs), and epoxy equivalent (EE). Each of these can be regarded as a measure of reaction progress, which was referred to as the % of reacted epoxide groups as well as % of reacted AA and expressed as epoxy group conversion (EGC) and acrylic acid conversion (AAC). The synthesized EA prepolymer was 98.4% solids (NV). The viscosity of the resin increased and is 70 Pas. The acid number of the product (PAVs) was 2.9 mgKOH/g, which indicates a conversion of acrylic acid in the amount of 97.9%. In turn, the EE was 408, so the conversion degree of epoxy groups was 49.6%. The molecular weight of the obtained prepolymers was 977 g/mol.

### 3.2. The Properties of Photocurable Coatings

The kinetics of photopolymerization is useful in understanding the rate and degree of curing. It is well known that acrylates and methacrylates do not polymerize by a cationic polymerization mechanism, while epoxy monomers do not polymerize by free-radical means. Hence, when cationic photoinitiators were applied, polyether-type polymer chains with pending (meth)acrylate groups were formed. In the case of free-radical polymerization, epoxy (meth)acrylates certainly formed a polymethacrylate backbone with pending epoxy groups [23]. The process of photopolymerization of the obtained resin was tested by both *photo* differential scanning calorimetry (photo-DSC) and Fourier transform infrared spectroscopy for real-time process monitoring (RT-IR) methods. In a first approach, the photoinitiated cationic or radical polymerization of the obtained EA prepolymer was monitored by means of the photo-DSC method. Photo-DSC offers a simple method of characterizing the UV-curing kinetics for the photopolymerization of UV-cured materials. Therefore, the profiles for the heat of reaction versus time provided by photo-DSC can be used to describe the photoinduced reaction kinetics and evaluate the polymerization rate. In turn, through the RT-IR method, the curves of the conversion of epoxy or acrylate groups and photopolymerization rate (Rp) of EAs compositions with various photoinitiators were investigated. As expected, the progressive disappearance of the various IR bands characteristic of the epoxy or acrylate double bonds was observed. The extent of the epoxy groups’ reactions was determined by the areas of the peak at 915 cm^−1^, which is due to C–O stretching in the epoxy ring. Simultaneously, the extent of double bond reactions was determined by the peak areas of the double bond peak at 1635 cm^−1^.

**Figure 8:**
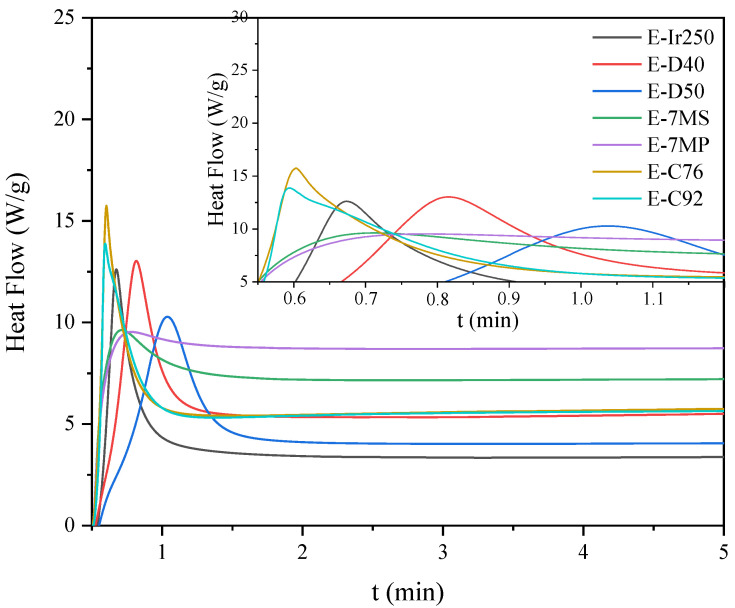
Photo-DSC exotherms for the photopolymerization of epoxy resin formulations with cationic photoinitiators.

**Figure 9:**
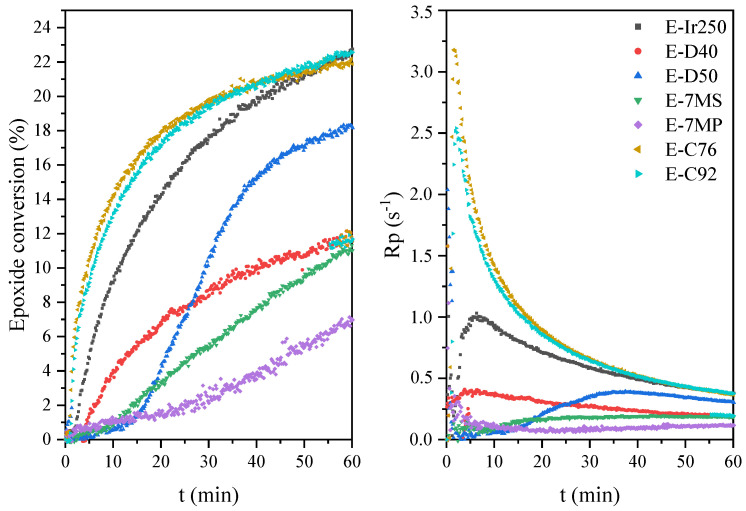
The epoxide conversion and photopolymerization rate curves of the epoxide resin formulations with cationic photoinitiators.

Owing to the presence of both epoxy and double carbon–carbon bonds in the prepolymer chain, the resulting EAs are expected to exhibit curing behavior via two distinct mechanisms: (i) cationic ring-opening polymerization or (ii) free radical polymerization. First, the kinetics of cationic photopolymerization of the mixture of unmodified epoxy resin (E) and various cationic photoinitiators (3 wt.%) were investigated. In the photo-DSC tests (irradiation conditions: 280–480 nm, 200 mW/cm^2^), it was shown that triaryliodonium salts polymerize the fastest (C76 and C92; absorption characteristics: 240 and 300 nm), despite their UV-C and UV-B absorption characteristics (Figure 8). They are followed by the diaryliodonium salts, i.e., (4-methylphenyl)[4-(2-methylpropyl)phenyl]iodonium hexafluorophosphate (Ir240), bis(dodecylphenyl) iodoniumhexaflouroantimonate (D40; absorption characteristics: 240 and 242 nm), and bis-((C10-C13)alkylphenyl)-iodoniumhexafluoroantimonate (D50; absorption characteristics: 240 and 242 nm), and the slowest were (7-methoxy-4-methylcoumarin-3-yl)phenyliodonium hexafluoroantimonate (7MS) and (7-methoxy-4-methylcoumarin-3-yl)phenyliodonium hexafluorophosphate (7MP; absorption characteristics: 350 nm). Similar results were obtained by the RT-IR method (irradiation conditions: 280–400 nm, 10 mW/cm^2^). Additionally, the conversion of epoxy groups was also investigated. As shown in Figure 9, the fastest polymerizing systems also had the highest degree of epoxy groups conversion. In the case of a relatively low polymerization rate, this type of relationship is often observed in literature reports.

**Figure 10:**
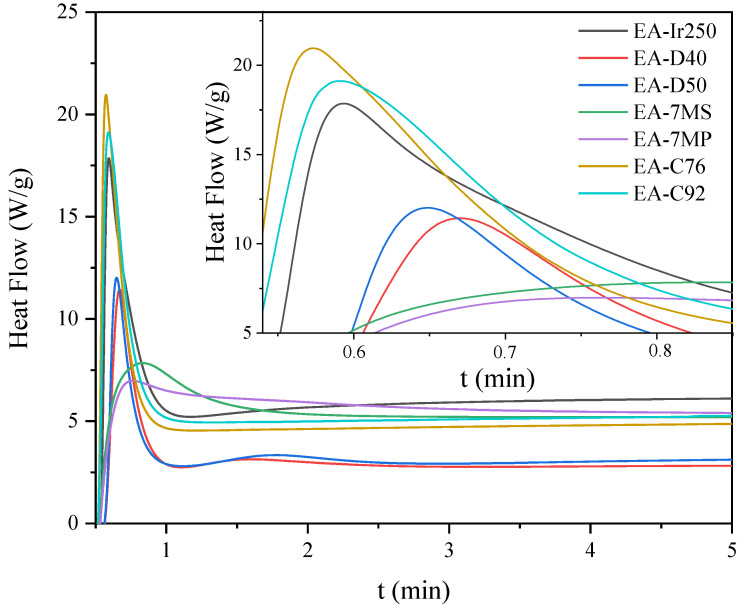
Photo-DSC exotherms for the photopolymerization of epoxy acrylate formulations with cationic photoinitiators.

**Figure 11:**
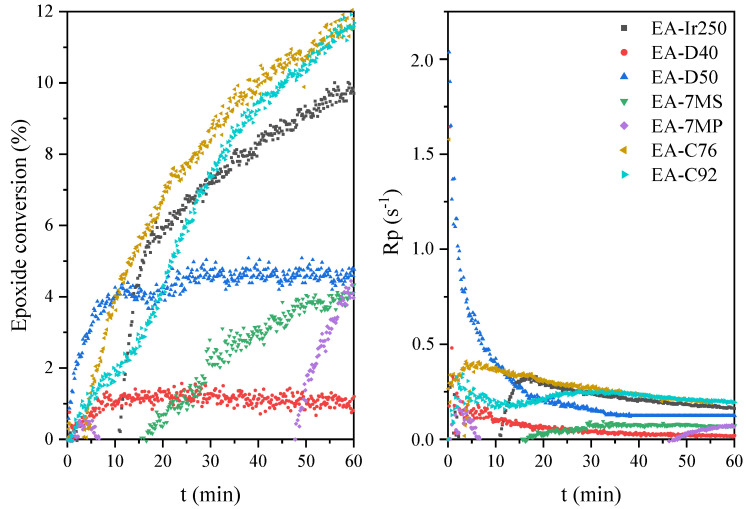
The epoxide conversion and photopolymerization rate curves of the epoxy acrylate formulations with cationic photoinitiators.

Further, the research on systems from the synthesized hybrid epoxy acrylate resin is presented. It is worth emphasizing that owing to the presence of both epoxy and double carbon–carbon bonds in the polymer chain, the resulting EAs are expected to exhibit curing behavior via two distinct mechanisms: (i) cationic ring-opening polymerization and (ii) free radical polymerization. In the first approach, the photoinitiated cationic polymerization of the EA prepolymers was monitored by means of the photo-DSC method. The compositions used in the study included synthetized EA and a cationic photoinitiators (3 wt.%). Figure 10 shows the photocalorimetric exotherms for the photoinitiated cationic polymerization of obtained mixtures. The tested systems polymerize at a similar rate as unmodified systems, i.e., the fastest systems with triaryliodonium salts, then the diaryliodonium salts, and some diaryliodonium salts (7MS and 7MP). However, as shown in Figure 11, epoxy acrylate prepolymers are characterized by obtaining a lower conversion of epoxy groups compared to the unmodified resin and a lower photopolymerization rate. This effect is directly related to the lower content of epoxy groups in these systems due to partial reaction of these groups with acid at the stage of EAs’ prepolymer synthesis.

**Figure 12:**
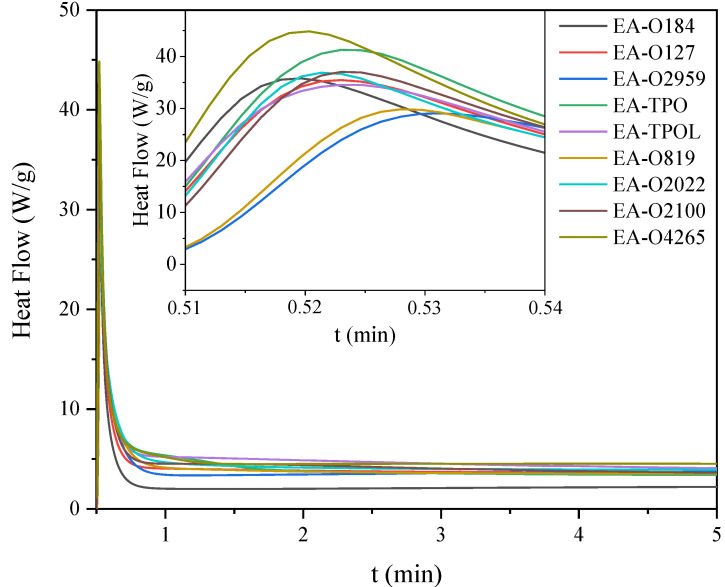
Photo-DSC exotherms for the photopolymerization of epoxy acrylate formulations with radical photoinitiators.

**Figure 13:**
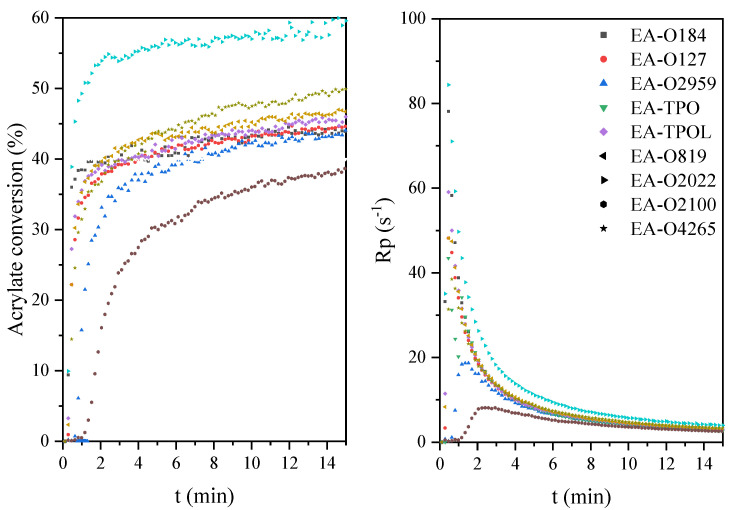
The acrylate conversion and photopolymerization rate curves of the epoxy acrylate formulations with radical photoinitiators.

The next part concerns photoinitiated free radical polymerization of acrylate groups of EA chains, which was also monitored by both photo-DSC (Figure 12) and RT-IR (Figure 13) methods. In this case, the compositions used in the research included synthetized EA and a radical photoinitiators (3 wt.%) belonging to α-hydroxyketones, acylphosphine oxides, and mixtures of them. The photocalorimetric exotherms show the course of photoinitiated radical polymerization of the mixtures obtained. The investigated systems polymerize faster than the cationically polymerizing systems. The EA-O4265 system polymerized the fastest, and the slowest is EA-O2959. The highest conversion of acrylate groups was characterized by systems with photoinitiators in the form of mixtures of α-hydroxyketones and acylphosphine oxides (EA-2022—60% and EA-O4265—50%), and these were much higher values compared to the conversion of epoxy groups in the cationic process. Thus, both the photopolymerization mechanism and the selection of an appropriate photoinitiator play an important role in the course of the process.

In order to determine the effect of the type (cationic or radical) and the course of photopolymerization of systems with different photoinitiators, the basic properties of the cured films were investigated. The results are presented in Table 2. First, the results concerning the properties of coatings obtained from epoxy resin with cationic photoinitiators are presented, followed by the coatings obtained from epoxy acrylate resin with cationic or radical photoinitiators.

Both coatings prepared from epoxy and epoxy acrylate resins obtained in the cationic process achieve the shortest tack-free time with use of certain photoinitiators in the form of diaryliodonium cation ((7-methoxy-4-methylcoumarin-3-yl)phenyliodonium hexafluoroantimonate (7M-S) and (7-methoxy-4-methylcoumarin-3-yl)phenyliodonium hexafluorophosphate (7M-P)). Although these photoinitiators lead to the lowest conversions of epoxy groups among all tested systems and run slower, their shortest tack-free time can probably be attributed to absorption in the longer wavelength range (350 nm), which is responsible for the surface drying of the coatings. In turn, the longest tack-free time has coatings with other diarylyodonium cations, whose absorption characteristics are at shorter wavelengths, i.e., in the range of UV-C radiation. It is well known that UV-C radiation penetrates deep into the coatings; therefore, it is difficult to obtain surface dryness. Epoxyacrylate coatings achieved a lower degree of conversion, and to obtain tack-free time coatings, they were cured for a longer time than epoxy coatings. Interestingly, however, EA coatings have a higher hardness compared to epoxy resin coatings. Despite the lower number of epoxy groups, epoxy acrylate resins should produce coatings of lower hardness than epoxy resins. Their higher hardness can be attributed to longer curing time, which is associated with cross-linking the polymer and greater post-curing of the coatings. It turns out that the choice of photoinitiator affects the adhesion. The coatings with triarylsulfonium cations showed the best adhesion. This may be related to the achievement of a high degree of conversion of the epoxy groups. All coatings obtained in the cationic process had a high gloss, and the highest yellowness was found in diarylyodonium cations, in particular 7MS and 7MP.

In comparison to the cationic process, the radical process of curing epoxy acrylate formulations was characterized by a much shorter time-free time, and most coatings with radical photoinitiators achieve it within 3 s (while in the cationic process, this time was achieved after 33 s). This is probably related to the much higher rate of radical polymerization compared to the cationic process. However, despite obtaining surface dryness in a short time, a higher photopolymerization rate, and much higher conversion of unsaturated bonds from acrylate groups, the coatings obtained in the radical process have lower hardness compared to those obtained in the cationic process. This effect may be related to the formation of flexible networks in the form of polymethacrylate backbone with pending epoxy groups, as opposed to the more rigid networks obtained by the cationic process, resulting in polyether-type polymer chains with pending unreacted acrylate groups. The stiffness of polyether networks may also result from the possibility of forming hydrogen bonds, which additionally stiffen the structure. It has also been shown that the selection of a radical photoinitiator affects the photopolymerization rate, conversion, tack-free time, and the properties of cured coatings, in particular, adhesion, gloss, and yellowness.

## 4. Conclusions

In this paper, an epoxy acrylate resin based on the industrial-grade bisphenol A-based epoxy resin and acrylic acid have been synthesized in order to develop hybrid resin comprising both epoxide group and reactive, terminal unsaturation. The significant advantage of this type of resin is the presence of two functional groups in one molecule, which can be used in the photopolymerization process and obtain coatings with a wide range of properties or used for subsequent modification. The obtained epoxy acrylate prepolymer was employed to formulate photocurable coating compositions containing, besides the EA binder, also cationic or radical photoinitiators. Owing to the presence of both epoxy and double carbon–carbon pendant groups, the reaction product exhibits photocrosslinking via two distinct mechanisms: (i) cationic ring-opening polymerization and (ii) free radical polymerization. The mechanism of photopolymerization, the type of the polymer network formed, and the selection of the photoinitiator have been shown to affect the kinetics of the process as well as the properties of the cured coatings. The cationic polymerization proceeds slower and leads to a lower degree of reaction compared to the radical process. As a result of this process, the surface dryness of the cured coatings is achieved in a longer time; however, the coatings have a higher hardness and gloss. It has also been shown that the kinetics of polymerization and the properties of cured coatings differ when using various types of photoinitiators. The properties of the coatings obtained by the cationic and radical processes may differ, among others, because they form different polymer networks. When cationic phtoinitiators were applied, polyether-type polymer chains with pending acrylate groups were formed. In the case of free radical polymerization, epoxy acrylates certainly formed a polyacrylate backbone with pending epoxy groups. The polyether-type polymers produce higher hardness coatings in relation to polyacrylates.

## Figures and Tables

**Table 1:** The maximum absorption peaks of the photoinitiators used.

Photoinitiator				Absorption Peaks (nm)
Type		Name	Abbreviation	
Cationic	diaryliodonium cation	Irgacure 250	Ir250	242
		Deuteron UV1240	D1240	240
		Deuteron UV1250	D1250	240
		Sylanto 7M-S	7MS	350
		Sylanto 7M-P	7MP	350
	triarylsulfonium cation	Cyracure UV6976	C76	240,300
		Cyracure UV6992	C92	240,300
Radical	α-hydroxyketones	Omnirad 184	O184	246,280,333
		Omnirad 127	O127	200,260
		Omnirad 2959	O2959	200,280
	acylphosphine oxides	Lucirin TPO	TPO	295,368,380,393
		Lucirin TPO-L	TPOL	300,380
		Omnirad 819	O819	295,370
	mixtures	Omnirad 2022	O2022	246,282,370
		Omnirad 4265	O4265	275,370
		Omnirad 2100	O2100	240,272,380

**Table 2:** Photopolymerization characteristics of the reaction mixtures and the basic properties of the cured coatings.

Sample	H_max_^(1)^ (W/g)	R_p_^max (2)^ (s^−1^)	X ^(3)^ (%)	Tack-Free Time (s)	Hardness (s)	Adhesion	Gloss (GU)	Yellowness Index
E-Ir250	13	1.1	22	33	324	2.5	160	6.9
E-D40	13	0.4	12	39	266	3.5	180	5.4
E-D50	10	0.4	18	39	194	2.5	170	5.6
E-7MS	9	0.2	11	30	118	3.5	115	21.6
E-7MP	9	0.4	6	30	244	2	163	21.9
E-C76	16	3.2	21	39	297	0	188	7.6
E-C92	14	2.5	22	33	234	0	187	7.5
EA-Ir250	18	0.3	10	57	333	1	89	6.7
EA-D40	11	0.2	1	57	310	3	100	4.5
EA-D50	12	2.0	5	57	205	3	127	3.7
EA-7MS	5	0.1	4	36	235	3	95	19.7
EA-7MP	4	0.2	4	39	267	4	163	15.7
EA-C76	21	0.4	12	39	344	1	170	6.3
EA C92	19	0.3	12	39	312	1	170	7.3
EA-O184	36	78.2	44	3	101	4	43	7.1
EA-O127	35	48.2	44	3	95	1	117	8.9
EA-O2959	29	18.7	43	6	65	2	22	6.8
EA-TPO	41	43.1	40	3	112	4	55	9.6
EA-TPOL	35	59.1	45	3	88	4	120	7.6
EA-O819	30	48.1	47	15	83	2	30	8.7
EA-O2022	37	84.4	60	6	92	0	108	8.9
EA-O2100	37	6.7	39	3	117	4	135	7.5
EA-O4265	45	38.5	50	3	91	2	150	7.2

^(1)^ maximum heat flow peak; ^(2)^ maximum polymerization rate determined by RT-IR method; ^(3)^ conversion determined by RT-IR method; –no curing.

## Data Availability

Not applicable.
